# Bilirubin: A Promising Therapy for Parkinson’s Disease

**DOI:** 10.3390/ijms22126223

**Published:** 2021-06-09

**Authors:** Sri Jayanti, Rita Moretti, Claudio Tiribelli, Silvia Gazzin

**Affiliations:** 1 Fondazione Italiana Fegato-Onlus, Bldg. Q, AREA Science Park, ss14, Km 163.5, Basovizza, 34149 Trieste, Italy; ctliver@fegato.it (C.T.); silvia.gazzin@fegato.it (S.G.); 2 Faculty of Medicine, University of Hasanuddin, Makassar 90245, Indonesia; 3 Molecular Biomedicine Ph.D. Program, University of Trieste, 34127 Trieste, Italy; 4 Neurology Clinic, Department of Medical, Surgical, and Health Sciences, University of Trieste, 34139 Trieste, Italy; moretti@units.it

**Keywords:** bilirubin, yellow players, disease-modifying therapy, Parkinson’s disease

## Abstract

Following the increase in life expectancy, the prevalence of Parkinson’s disease (PD) as the most common movement disorder is expected to rise. Despite the incredibly huge efforts in research to find the definitive biomarker, to date, the diagnosis of PD still relies mainly upon clinical symptoms. A wide range of treatments is available for PD, mainly alleviating the clinical symptoms. However, none of these current therapies can stop or even slow down the disease evolution. Hence, disease-modifying treatment is still a paramount unmet medical need. On the other side, bilirubin and its enzymatic machinery and precursors have offered potential benefits by targeting multiple mechanisms in chronic diseases, including PD. Nevertheless, only limited discussions are available in the context of neurological conditions, particularly in PD. Therefore, in this review, we profoundly discuss this topic to understand bilirubin’s therapeutical potential in PD.

## 1. Introduction

Discovered as a rare disorder in 1817 by James Parkinson, nowadays Parkinson’s disease (PD) is evolving as the fastest growing neurological disorder and one of the leading causes of disability in the world [1,2]. Since the improvement of health care is followed by the world’s aging population, PD is estimated to be a non-infectious pandemic, with the number of people affected predicted to double from 6.9 million in 2015 to 14.2 million in 2040 [2]. Besides the increase of prevalence, years lived with the disability will be followed by increased different outcomes, including personal, social, and economic burdens that make PD research highly important [3,4].

The significant motoric symptoms, tremor, rigidity, and bradykinesia are the consequences of progressive and selective diminished dopaminergic neurons in the substantia nigra pars compacta (SNc) [5,6]. Moreover, reliable data on the presence of non-motoric symptoms (hyposmia, psychiatric symptoms, rapid eye movement sleep behavior disorder, dementia, pain, fatigue, constipation) have established that PD is not exclusively due to dopaminergic neuron loss but also involves non-dopaminergic neurons [6]. Although the etiology of PD has not been firmly established yet, advanced age, male sex, environmental factors (e.g., toxins and pesticides), and genetic traits have been recognized as relevant risk factors [4,7,8]. Multiple mechanisms, including mitochondrial dysfunction, oxidative stress, neuroinflammation, and proteostasis disturbances, are increasingly appreciated as key determinants of dopaminergic neuronal susceptibility in PD and are the feature of both familial and sporadic forms of the disease [9,10,11]. 

Current treatments for PD mainly rely on symptomatic treatments by administrating L-DOPA, dopamine agonists, inhibitors of dopamine-degrading enzymes, or neuroablative surgery. Nevertheless, none of these treatment regimens can prevent disease progression [12,13]. Moreover, undesirable side effects are present in the treatment such as those mentioned above (Table 1). Thus, the exploration of novel therapy addressing the disease progression and improving or delaying disability is needed. Meanwhile, bilirubin, the heme catabolic end product and long known as a toxic yellow pigment, has emerged with its plethora of therapeutic potentials. The protective role of bilirubin is suggested in Gilbert’s syndrome (a condition of mildly elevated bilirubin) subjects toward non-neurological conditions including cardiovascular disease, cancer, and metabolic syndrome [14]. In recent decades, mounting evidence has shown that bilirubin and its enzymatic properties (heme oxygenase and biliverdin reductase, known as yellow players) possess antioxidant and anti-inflammation qualities and are even involved in signaling pathways in a wide range of conditions, including neurodegenerative diseases [14,15,16]. However, little discussion is present regarding its protective effect in PD. This review addresses the role of bilirubin as a potential disease-modifying therapy for PD.

## 2. Parkinson’s Disease: From Pathogenesis to Management

### 2.1. Pathogenesis

**Figure 1:**
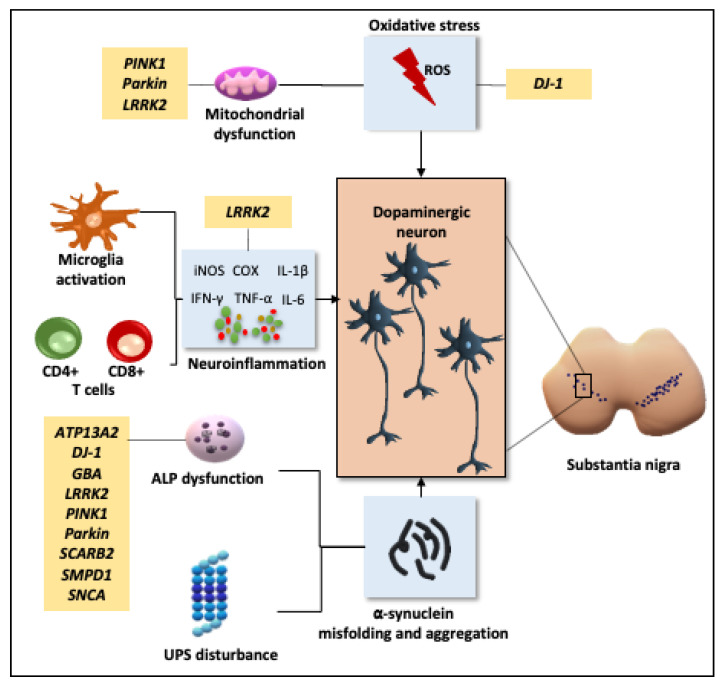
Involved mechanism in pathogenesis of Parkinson’s disease. Multiple mechanisms known to be involved in the pathogenesis of dopaminergic neuron loss including oxidative stress, neuroinflammation, α-synuclein misfolding and aggregation, and genetic influence. Abbreviation: ALP, autophagy lysosomal pathway; UPS, ubiquitin proteasome system; iNOS, inducible nitric oxide synthase; COX, cyclooxygenase; TNF-α, tumor necrosis factor-α; IFN-γ, interferon-γ; IL, interleukin -6 and -1β; *DJ1*, Daisuke-Junko-1; *Pink1*, acid protein phosphatase and tensin homolog (PTEN)-induced kinase 1; *ATP13A2*, ATPase type 13A2; *MAPT*, microtubule-associated protein tau; *GBA*, glucocerebrosidase; *SNCA*, α-synuclei; *LRRK2*, Leucine-rich repeat kinase 2; *SMPD1*, acid-sphingomyelinase; *SCARB2*, scavenger receptor class B member 2.

Although the pathogenesis of PD remains to be fully elucidated, many lines of evidence including postmortem, in vitro, and animal model studies unraveled the involved mechanisms of PD (depicted in Figure 1). These include:

#### 2.1.1. Oxidative Stress and Mitochondrial Dysfunction

Due to its high consumption of oxygen, extensive production of reactive oxygen species (ROS), and low level of antioxidant enzymes, the brain is vulnerable to oxidative stress [32,33]. The involvement of oxidative stress in PD has been explored in postmortem analysis with the detection of an increased amount of lipid peroxidation markers, carbonyl modification of soluble proteins, and DNA damage [34,35]. Furthermore, clinical evidence showed the presence of oxidative stress markers in blood and cerebrospinal fluid [36]. In experimental settings, the link between oxidative stress and dopaminergic neuron degeneration has been confirmed. Oxidative stress not only has a direct effect on cellular damage but also influences the activation of signaling pathways leading to cell death [37]. 

Mitochondrial dysfunction is closely connected to the increased ROS formation in PD. ROS production is physiologic and powers neural activity and maintains cellular homeostasis. However, mitochondrial dysfunction, especially in the electron transfer chain, can lead to excessive ROS production which is detrimental to cells [33]. On the other hand, ROS also drives further harm to the electron transport chain itself [38]. The deficiency and impairment of mitochondrial complex-I activity, the vital component of the electron transport chain, was found in postmortem studies and dopaminergic-cell-loss-induced animals by toxin and pesticides [39,40]. The defects of mitochondrial complex-I of the respiratory system lead to degeneration of neurons due to lack of ATP production [37].

The reasons of dopaminergic neuron SNc vulnerability to mitochondrial dysfunction have been hypothesized to be related to (i) the size and complexity larger than other types of neurons in the brain demanding high rates of ATP production to keep resting membrane potential, induce action potential, and allow synaptic transmission, (ii) distinctive physiology of action potential which distinguishes SNc dopaminergic neuron from the majority of neurons in the brain, and (iii) the reliance upon dopamine as a neurotransmitter which is considered as a potentially toxic compound if accumulating into the cytosol [11,41,42]. 

#### 2.1.2. Neuroinflammation

Neuroinflammation is one of the main features of PD that has been shown in clinical studies as well as experimental settings [43,44,45]. Microglia activation seems to be the primary mediator for the inflammatory process in PD. Microglia have been documented to initiate inflammatory response in PD [10,46]. The activation of microglia is due to α-synuclein, a danger-associated molecular pattern (DAMP), which can directly trigger microglial activation and initiate sterile inflammatory processes [47,48,49]. For instance, in primary cultures, α-synuclein mediates dose-dependent activation of microglia [50].

α-synuclein is not the only stimulant for microglia activation as multiple agents have been demonstrated to have a microglia activator effect, including debris of degenerating neurons [51]. Additionally, the microglial activation is significantly exacerbated by not only rotenone treatment but also lipopolysaccharides (LPS), indicating multiple mechanisms responsible for microglia activation [52,53]. Shor-term activation of microglia provides neuroprotection, whereas long-term activation leads to the neurodegeneration process. Noteworthy, activated microglia have been demonstrated as a critical ROS source, further indicating that the inflammation process induces oxidative stress and vice versa.

Microglia activation promotes the activity of pro-inflammatory enzymes (such as inducible nitric oxide synthase (NOS) and cyclooxygenase (COX)) and the release of the pro-inflammatory cytokines, such as C-X-C motif chemokine ligand 12 (CXCL12), tumor necrosis factor-α (TNF-α), interferon-γ (IFN-γ), and interleukin (IL)-6 and IL-1β [54]. Moreover, the inflammatory responses of microglia are amplified by astrocyte senescence in the aging brain [55,56]. 

Additionally, the involvement of the adaptive immune system has been observed in PD through the presence of CD8+ and CD4+ T cells in the brain in both postmortem human PD specimens and the MPTP mouse model [57]. This conclusion has been supported by Sulzer et al. who found that α -synuclein peptides acted as antigenic epitopes and induced T cell response in PD patients [58]. Finally, a longitudinal study showed that in PD patients, a more “pro-inflammatory” component profile (TNF-α, IL1-β, IL-2, and IL-10) in the serum is associated with a faster motor syndrome progression and more cognitive decline [59]. 

#### 2.1.3. Disruption of Cellular Proteostasis

Protein clearance is an intracellular defense mechanism to ensure protein homeostasis by rapid detection of altered protein and its elimination [60]. Molecular chaperones, the ubiquitin-proteasome system (UPS), and the autophagy-lysosomal pathway (ALP) are the essential pathways that facilitate the clearance of abnormal proteins [61,62]. UPS selectively shatters short-lived proteins and misfolded or damaged intracellular proteins, whereas ALP degrades the longer-lived proteins, cellular components, and organelle through the lysosomal compartment [63,64]. The proteasome dysfunction exacerbates protein aggregates in PD. Convincing evidence showed that α-synuclein deposition, which later becomes Lewy body inclusion in PD subjects, is the consequence of the failure of those degradation pathways [63]. The decrease in UPS activity has been explicitly reported in the substantia nigra of the PD brain [65,66]. It has been demonstrated that the presence of misfolded protein UPS failure in dopaminergic neurons was induced by the expression of mutant α -synuclein [67]. 

#### 2.1.4. Genetic Influence

Although the familial forms of PD account for only 5–15% of the cases, these cases have offered essential insights regarding genetic influences in PD pathogenesis [68]. The genetic changes play a role in molecular pathways, including α-synuclein proteostasis and degradation, mitochondrial function, oxidative stress, and neuroinflammation. Mutation in *DJ1* (Daisuke-Junko-1), *Parkin*, *Pink1* (acid protein phosphatase and tensin homolog (PTEN)-induced kinase 1), and *ATP13A2* (ATPase type 13A2) is responsible for monogenic PD forms, and other genes, including *MAPT* (microtubule-associated protein tau), *GBA* (glucocerebrosidase,), *APOE* (apolipoprotein E), have been associated with an increased risk of developing PD. Meanwhile, *SNCA* (α-Synuclein) and *LRRK2* (Leucine-rich repeat kinase 2) play a role not only in monogenic form but also as risk factors of PD [69,70]. 

The mutation in *SNCA*, the gene encoding for α-synuclein, has been known as the cause of heritable forms of PD by leading to α-synuclein dysfunction and aggregation [71]. Moreover, single nucleotide polymorphism in this gene is associated with the risk for sporadic form [72]. Several genetic mutations related to autophagy lysosomal pathway including *LRRK2*, *GBA*, *SMPD1* (acid-sphingomyelinase), *SNCA*, *PINK1*, *Parkin*, *DJ1*, and *SCARB2* (scavenger receptor class B member 2) are involved in PD [70]. Some of these genes encode lysosomal enzymes, whereas others correspond to proteins involved in transport to the lysosome, mitophagy, or other autophagic-related functions [73]. Mutations in *PINK1* and *Parkin* involve mitophagy impairment, accelerate the accumulation of defective mitochondria, and lead to dopaminergic neuron loss [74]. Mutations in *DJ1*, a gene that encodes a putative antioxidant, are related to enhanced oxidative stress. Recent advances in understanding the genetic influence in PD have uncovered that a gene mutation can be related to multiple molecular pathways. For example, *LRRK2* mutations are not only associated with autophagy and lysosomal degradation but also neuroinflammation, mitochondrial dysfunction, and neurotransmission [75]. 

### 2.2. Challenges in the Management of PD

Despite the numerous efforts directed toward diagnostic modality, the diagnosis of PD still rests on clinical manifestation. Since the prodromal symptoms of PD are non-specific, including rapid eye movement, sleep behavior disorder, loss of smell, constipation, urinary dysfunction, orthostatic hypotension, excessive daytime sleepiness, and depression, most PD patients get diagnosed after the cardinal motor symptoms appear. The motor manifestations become apparent after dopaminergic neuron loss in the substantia nigra pars compacta reaches about 40–50% [76]. 

The most used therapy is mainly to replace and boost the existing dopamine. Levodopa, the dopamine precursor, is most frequently used to alleviate motor symptoms. It is usually combined with carbidopa for blocking its metabolism in the periphery and increases its bioavailability in the central nervous system [77]. Levodopa offers significant symptomatic advantages, but its long-term use is followed by motor complications (dyskinesia and motor fluctuation) [78]. Dopamine agonists (pramipexole, ropinirole, and rotigotine), the stimulants for dopamine receptors in the central nervous system, are suitable in the management of mild to moderate PD. However, the side effects such as orthostatic hypotension, hallucinations, confusion, leg edema, and impulsive disorder have been frequently reported in individuals under dopamine agonist therapy [17,79]. Catechol-O-methyl transferase inhibitors (entacapone) and monoamine oxidase aldehyde dehydrogenase B (MAO-B) inhibitors (rasagiline and selegiline) inhibit enzymes involved in the breakdown of levodopa and dopamine [80]. Anticholinergic medications (trihexyphenidyl and benztropine) are not effective in treating bradykinesia but may decrease rigidity, dystonia, and tremor. For the young individual, caution is a must because of the potential of adverse events, particularly relating to cognition [80]. 

Deep brain stimulation targeting either the subthalamic nucleus or globus pallidus internus has evolved as an important therapy for PD and is usually performed in a relatively early-onset patient [81,82]. Despite deep brain stimulation being considered as well tolerated, complications due to the surgical techniques such as intracranial hemorrhage, high chance of re-surgery caused by hardware-related complications or infections, and microlesion effects due to electrode penetration (that affect cognitive states and psychiatric conditions) are reported [28]. 

Cell therapy by using pluripotent stem cells, including human embryonic stem cells (hESCs) or induced pluripotent stem cells (iPSCs), is emerging as a novel experimental approach to tackle this problem. iPSCs are preferable over hESCs due to similar differentiation potential but fewer ethical concerns [8]. Interestingly, iPSCs have been explored as personalized medicine in PD because of their autologous entity (gained from a patient donor), which lowers the chance of graft rejection [30,83]. However, several issues present in the application of iPSCs, including the risk of tumor formation as in hESCs implantation and the heterogeneity of iPSCs due to the genetic modification, variable transgene expression levels, incomplete reprogramming, and reactivation/lack of inactivation of the transgenes. Moreover, PD-patient-derived iPSCs may carry mutations and could be susceptible to developing PD-like features [29,30]. These issues might be corrected by using genomic editing, particularly CRISPR-Cas9, as performed in the iPSCs PD model [84]. 

So far, it is undeniable that all the aforementioned pharmacologic and non-pharmacologic approaches can reduce motoric symptoms by targeting the remaining dopaminergic neuron to produce more dopamine, stimulating the dopaminergic receptor, inhibiting the breakdown of dopamine, or replacing dopaminergic neurons. However, none of the intervention strategies mentioned above has a disease-modifying effect of encountering the molecular mechanism involved in PD pathogenesis [6,80]. 

The lack of experimental models that mirror the phenotypic manifestation of PD is the main limitation to understand the disease pathophysiology and translate the therapy to the patient. None of the available animal models, from neurotoxic models to genetic models, perfectly represent the neuropathology of PD and mimic the clinical syndrome. Despite the ability of neurotoxin (e.g., MPTP (1-methyl-4-phenyl-1,2,3,6-tetrahydropyridine) and 6-OHDA (6-hydroxydopamine)) and transgenic models to unravel the pathological mechanisms, current animal models are limited by the incapability to replicate the dopaminergic neuron loss and formation of α-synuclein in a single model at the same time. Nevertheless, the wide variety of animal models (rodent, non-human primates, and non-mammalian models) allows targeted studies of different pathological mechanisms of PD [85]. 

## 3. Bilirubin and the Yellow Players in Neurological Diseases

**Figure 2:**
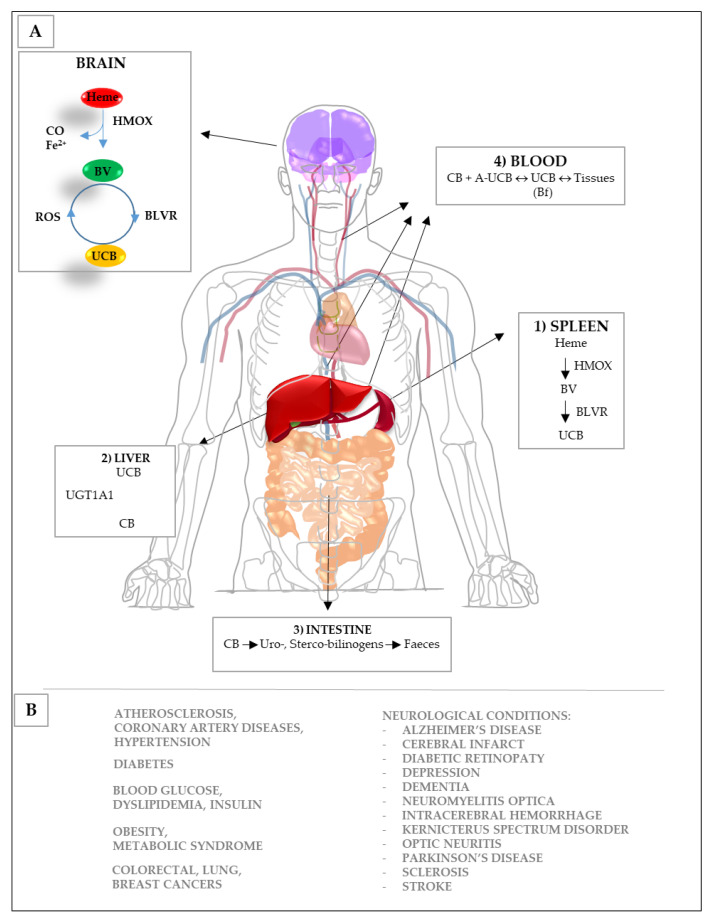
Bilirubin catabolism and biological functions. (**A**) In the spleen (**1**), hemoglobin (Heme) derived from the red blood cells, is converted to unconjugated bilirubin (UCB) by the action of heme-oxygenase (HMOX) and biliverdin reductase (BLVR; BV = biliverdin), thus vehicolated to the liver by blood. (**2**) In the liver, the lipophilic UCB is converted to the hydrophilic conjugated bilirubin (CB, by the uridine-diphospho glucuronosyl transferase: UGT1A1), thereafter excreted in the intestine (**3**), where it is transformed in the uro- and sterco-bilinogens before excretion. Bilirubin in blood (**4**) is present as: CB (or direct bilirubin, the minor part) and UCB (or indirect bilirubin), constituting the major fraction in physiological conditions. In turn, blood UCB is present in two forms: bound to albumin (UCB-A, the 99% of circulating UCB in physiological conditions) and the unbound UCB (the so-called free bilirubin: Bf) [96]. The sum of CB and UCB form the total serum bilirubin (TSB), routinely quantified in the clinic. Because of the molecular size of the complex, UCB-A is confined in the vascular lumen, while the small lipophilic Bf easily diffuses across the cellular bilayers entering (in equilibrium with) the tissues [97,98]. HMOX, BV, BLVR, and UCB (the yellow players: YP) are present also in the brain, where they have been hypothesized to act as a potential defensive mechanism toward neurological conditions by reacting on-demand to stressor/pathologic stimuli and producing UCB in situ. (**B**) Based on epidemiological and experimental data, a minimal increase in the bilirubin level has been suggested to be benefic toward both extra-CNS and CNS chronic diseases acting as an anti-oxidant; on immunity and inflammation; on the cell cycle, proliferation, differentiation, migration, and apoptosis; and controlling glucose and insulin homeostasis [14,15,86,87,88,89,90,91,92,93,94,95].

Bilirubin is the yellow product of hemoglobin catabolism (see Figure 2A), clinically known as a serum marker of hepatic diseases. Recently, bench-based and epidemiological data point to a health-promoting effect of the pigment toward chronic diseases (Figure 2B) (reviewed in [14,86]). 

A new interesting perspective in the context of CNS pathologies has also emerged. Recent data reported the expression and activity of the enzymes involved in UCB production (the yellow players (YPs): HMOX, heme oxygenase 1,2; BV, biliverdin; BLVR, biliverdin reductase A (Figure 2A)) in the CNS, macking the brain able to produce UCB independently from the blood supply. Importantly, the in situ UCB production has shown increasing cellular resistance to damage [87,88,89]. Moreover, both HMOX1 and BLVRA, the two key enzymes in UCB production, possess multiple binding sites for transcription factors on the promoter region of the gene, making them promptly inducible by a wide range of stressor stimuli, including those characterizing the neurological conditions [15,90,91,92,93,94]. Finally, each YP may act directly or indirectly (through signaling pathways) on key biological functions, expanding the potential for protection [95]. These features collectively make the YPs a homeostatic and defensive system that enhances the capability of a neuronal cell to protect itself under a stress condition or even making the CNS independent from the serum UCB level.

### 3.1. Potential Mechanisms of Action

The study of YP neuroprotection toward PD is at its beginning. Nevertheless, some preliminary interesting information on YP protective action on the pathological mechanisms of ongoing PD may be extrapolated from the literature (Table 2).

#### 3.1.1. Oxidation

Since the 1980s, UCB (or indirect bilirubin in clinical words) is known as the most powerful endogenous antioxidant [95,96], mostly accounting for the preferential scavenging of lipophilic radicals that can attack lipid membranes [97,98,99]. During the ROS/RNS (reactive oxygen/reactive nitrogen species) scavenging activity [100,101,102], UCB is oxidized back to BV and in turn rapidly reconverted to UCB by the BLVRA. As a result, nano-molar concentrations of UCB can neutralize 10,000 times higher levels of cellular ROS [97,103], without increasing the UCB cellular level to a toxic concentration (Figure 2A). In cell cultures, UCB has been shown to activate the anti-oxidant response genes [104,105]. Moreover, it promotes additional cellular defense against redox stress. Indeed, by reducing ROS, UCB may inhibit the NMDA excitotoxicity, preventing neuronal death [106]. Anti-oxidant defenses (SOD and HMOX1), as well as the protective neuroglobin expression which reduces mitochondrial dysfunction, cytochrome C release, and apoptosis [106,107], and ferritin synthesis (chelating iron) [108] are also induced by intracellular free heme. In addition, BV may scavenge RNS [104], lowering DNA damage [108,109] and inhibiting lipid peroxidation with an efficacy 2-fold higher than α-tocopherol [105]. As a result, glutamate excitotoxicity [110], inflammation [111], and cell death by apoptosis [109] are reduced. Despite not being studied in the CNS context, BLVRA may also contribute to cellular protection by migrating into the nuclei and acting as a transcription factor on the genes involved in the cellular antioxidant response, immunity, and inflammation, autophagy, and apoptosis, hypoxia, tumor resistance, etc. [14,105].

On the other side, the rapid conversion of BV to UCB [112], accumulation of heme and iron (by excessive activation of HMOX1) may lead to brain dysfunction by including a cell energy failure, increased ROS/RNS production and DNA damage, inhibition of the antioxidant defenses, gliopathy, present in many aging-related neurodegenerative brain disorders, glutamate neurotoxicity, and cell death [15,91,108,113,114,115,116,117,118,119,120,121].

#### 3.1.2. Inflammation

Inflammation is the second critical pathological mechanism known to be modified by the YPs. Cytokine production is induced by heme, UCB, and iron (IL8, TNF-α), inducing the neutrophil migration, vascular permeability and edema [116,117], and ER stress [122], activating the microglia [117], and finally reducing the cellular viability [122,123,124].

On the other side, BVLRA (acting as a transcription factor), UCB, and CO may inhibit inflammation (TNF-α, Il6, complement, and T cell response) and inhibits NOS, diminishing endothelial cell apoptosis and preventing the alteration of the blood–brain barrier (BBB) [96,103,111,125,126,127,128,129,130,131,132]. 

**Table 2:** Experimental evidence of YP protective action on the pathological mechanisms in ongoing PD.

Pathological Mechanism in PD	YPs (Protective Effect)	Ref.
REDOX	UCB (↓) Heme (↓) BV (↓)	[95,96,97,103] [106,107,108] [104]
Anti-oxidant enzymes	UCB (↑) Heme (↑) BLVRA (↑)	[104,105] [106,107] [14,105]
Carbonylation and lipid peroxidation	Membrane protection by scavenging lipophilic radicals (↑) BV (↓)	[97,98,99] [105]
DNA damage	BV (↓)	[108,133]
Mitochondrial disfunction	Heme (↓) Heme: cofactor for the mitochondrial electron transport chain (complexes II, III, IV)	[106,107] [118]
PINK1/DJ1; LRRK2; SNCA; PARK2	No direct experimental data are yet available. Further, devoted studies are needed.	
INFLAMMATION	BV (↓) BLVRA, UCB, CO (↓) HMOX1 (↓)	[111] [96,103,111,125,126,127,128,129,130,131,132] [134,135]
Microglia and astrocyte activation	No direct experimental data are yet available. Further, devoted studies are needed.	
α-synuclein		
iNOS and COX	BLVRA, UCB, CO (↓)	[96,103,125,126,127,128,129,130,131,132]
TNFα	BLVRA, UCB, CO (↓)	[96,103,125,126,127,128,129,130,131,132]
IL6	BLVRA, UCB, CO (↓)	[96,103,125,126,127,128,129,130,131,132]
IL1β; IFNγ ; Il2; IL10; CXCLY2	No direct experimental data are yet available. Further, devoted studies are needed.	
CD8+ and CD4+ T cells	BLVRA (↓) BLVRA, UCB, CO (↓)	[14,105] [96,103,125,126,127,128,129,130,131,132]
LRRK2; SNCA	No direct experimental data are yet available. Further, devoted studies are needed.	
PROTEIN DEGRADATION		
UPS	No direct experimental data are yet available. Further, devoted studies are needed.	
Autophagy	BLVRA (↓)	[14,105]
LRKK2; GBA; SMPD1; SNCA; PARK2; PINK1/DJ1; SCARB2	No direct experimental data are yet available. Further, devoted studies are needed.	
GLUTAMATE TOXICITY	UCB (↓) BV (↓)	[106] [110]

↑: increased; ↓: decreased; UCB: unconjugated bilirubin; Heme: hemoglobin; BV: biliverdin; BLVRA: biliverdin reductase; PINK7/DJ1: acid protein phosphatase and tensin homolog (PTEN)-induced kinase 1; LRRK2: leucine-rich repeat kinase 2; SNCA: α-synuclein; PARK2: Parkinson juvenile disease protein 2; CO: carbon monoxide; HMOX1: heme oxygenase 1; iNOS: inducible nitric oxide synthase; COX: cyclooxygenase; TNFα: tumor necrosis alpha; IL: interleukin; CXCL12: C-X-C motif chemokine ligand 12; UPS: unfolded protein response; GBA: glucocerebrosidase; SMPD1: acid-sphingomyelinase; SCARB2: scavenger receptor class B member 2.

#### 3.1.3. The YPs in Parkinson’s Disease (PD)

A two-phase HMOX1/UCB modulation, the early induction interpreted as tentative protection toward the ongoing oxidative stress, and the late phase as the failure of the protection are hypothesized. Several pieces of evidence are available (Table 3). 

(1) YP induction in autopsy from PD patients has been described. Increased HMOX1 reactivity has been found in the dopaminergic neurons (DOPAns), microglia, and astroglia of the substantia nigra (SN) and in neurons of the neo-cortex presenting Lewy bodies [136]. Since HMOX1 is a redox sensor and an activator of the anti-oxidant response genes, the up-regulation of HMOX1 in the site of the lesions in PD has been suggested to belong to an early tentative reaction toward the ongoing redox imbalance by the in situ production of UCB. (2) In agreement with the protection, in rodent and in vitro models, the induction of HMOX1 has been correlated with a decreased inflammation and increased DOPAn survival [134,135]. Interesting for potential diagnostic applications is the correlation reported between the clinical stage of PD, the serum level of bilirubin (TSB), and HMOX1 induction. (3) Based on the data obtained by Lee [134] and Macias-Garcia [137], higher TSB (as well as its precursor BV [138]) is present at the onset of PD, together with an increased presence of UCB degradation products in the urine of PD patients, suggesting the induction of HMOX1 [139]. (4) Notably, patients with higher TSB present less severe symptoms and need less L-DOPA administration [140]. (5) Patients receiving L-DOPA, which is able to improve the symptoms of PD, have significantly higher TSB vs. both drug-naive PD and controls, suggesting that L-DOPA might reduce the redox imbalance, allowing HMOX1 to produce enough bilirubin for alleviating the disease [141]. (6) Supportive are also the recent findings correlating genetic polymorphisms on HMOX genes with PD incidence. Genetic variants on the HMOX1 gene, leading to its decreased transcription and inducibility (thus reduced UCB/BV production), and HMOX2 (the neuronal constitutive form) have been noticed to be significantly more frequent in subjects with the disease. Specifically, for the HMOX1 variants, a correlation with a more early onset of the symptoms has been reported [136,142]. These data may support the potential protective role of increased UCB production in the brain. However, the cited paper did not report the TSB level in the PD and control groups. Based on the described possible role as an early marker of PD, the increase of HMOX1 in saliva has been proposed as an easy-to-do, non-invasive marker of this neurodegenerative condition [143].

On the other hand, (7) the up-regulation of HMOX1 has been clinically documented to increase in situ iron deposition, enhance pro-oxidant milieu, and finally, worsen the damage [144,145,146]. The data have been supported by experimental models, where the excessive HMOX1 induction not only increased the iron deposition in the CNS but has also been suggested to enhance the oxidation of L-DOPA, an additional highly pro-oxidant molecule [147,148]. (8) As suggested for Alzheimer’s disease, under a condition of extreme redox stress, BLVR might be inactivated, becoming unable to foster the brain with UCB and eventually leading to failure in protection [149]. (9) In agreement, in the clinical setting, the TSB level in PD patients decreases with the increase in the severity of the disease. This negative correlation has been interpreted as the failure of the tentative defense with the consumption of UCB [137]. 

The unraveling of the interplay of YPs with PD progression is currently impossible in the clinical setting due to the late (symptom-based) diagnosis. The confirmation of what was described above and the dissection of the causative mechanisms from the consequential ones require experimental models able to mimic the time course of the disease from the pre-clinical stages, through the stages corresponding to an early diagnosis, to severe disease. Recently, an ex vivo model of DP reproducing the whole disease progression in 96 h has been developed by challenging brain organotypic cultures of substantia nigra with rotenone, a pesticide responsible for PD in the 1980s. This model confirms HMOX1 as one of the first genes up-regulated in PD (3 h, together with Tnfα and Cox2). The HMOX1 modulation precedes even the DOPAn demise usually detected at the diagnosis in PD patients (−40% vs. controls, 24 h), supporting the potential of the clinical use of HMOX1 as a diagnostic tool [45]. Further use of the model might allow demonstrating the effects of an increased UCB in protection toward DOPAn loss. 

**Table 3:** Evidence of YP modulation in PD.

YPs	Modulation	Ref.
HMOX1	(↑) In DOPAn, microglia, and astroglia of the SN. (↑) In neurons of the neo-cortex with Lewy bodies. (↑) In in vitro model of PD. Genetic variants of HMOX1 (leading to a reduced transcription and induction of the gene) are more frequent in PD subjects and correlate with an early onset of the disease.	[136,144,145,146] [136] [134,135] [136]
HMOX2	Genetic variants of the neuronal constitutive HMOX2 (leading to a reduced transcription) are more frequent in PD subjects.	[145]
TSB	(↑) In early clinical stages of PD. (↑) In PD patients with less severe symptoms. (↓) In late/more severe clinical stages of PD.	[140,150] [140] [137,150]

↑: increased; ↓: decreased; HMOX: heme oxygenase; DOPAn: dopaminergic neurons; SN: substantia nigra; PD: Parkinson’s disease; TSB: total serum bilirubin.

## 4. Future Prospective: Bilirubin as a Treatment in PD and Its Modulatory/Delivery System

**Figure 3:**
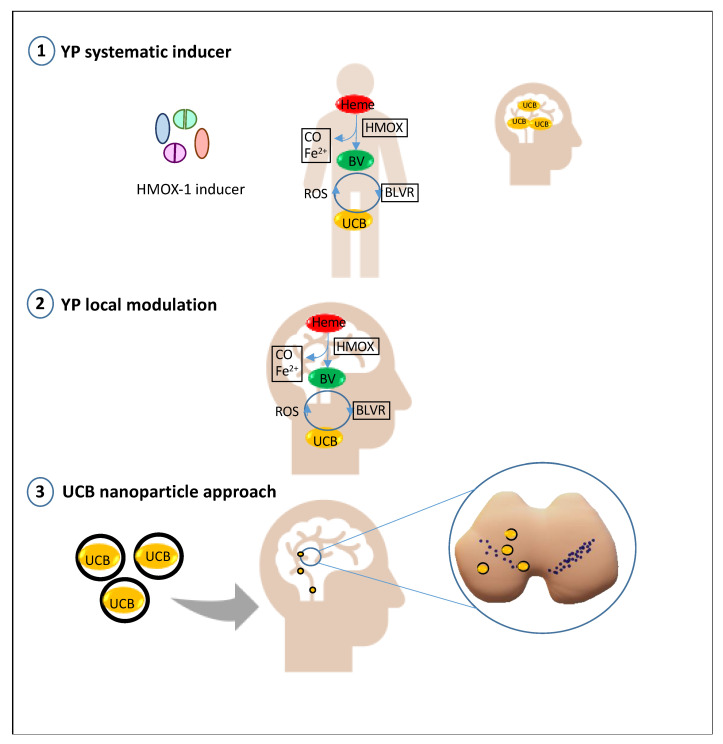
Illustration of possible bilirubin modulatory/delivery system in the brain. YPs: yellow players; HMOX: heme oxygenase; BLVR: biliverdin reductase; UCB: unconjugated bilirubin; ROS: reactive oxygen species.

From the general knowledge about UCB and the specific information in PD, two points seem clear: (a) UCB may protect if given in the non-toxic range, and (b) HMOX1 hyperactivation looks inevitable and worsens the ongoing damage. The critical point is how to reach the correct (protective) amount of UCB avoiding the hyperactivation of HMOX1 (Figure 3).

A pharmacological approach targeted in a systemic (whole-body) modulation of the YPs seems to be the most obvious and is already primarily evaluated in extra-CNS diseases [151,152,153] by inducing HMOX1 and increasing TSB. 

A second approach might consist of modulating the YPs directly in the CNS. This approach seems limited because HMOX1 is already induced at the time of the diagnosis. Thus, inducing even more HMOX1 will possibly enhance the side effects and accelerate the disease progression. 

A third exciting alternative might be the use of nanoparticles. Nanoparticles designed explicitly for brain delivery (of mRNA, small peptides, chemotherapeutic agents, etc.) are under evaluation [154,155,156,157,158] and are of routine clinical use as agents in magnetic resonance, magnetic-field-directed drug targeting to tumors across the blood–brain interfaces (BBIs), and for direct anti-tumor treatment by magnetic hyperthermia (ref. in [155,156,157,158,159,160]). The potential ways of administration are both invasive (e.g., intracranial, after the temporary opening of the BBI), marginally invasive (i.p. in animals; i.v.), or non-invasive (nasal route) [155,157]. Size and charge, selection of the material for the scaffold, engineering of the particles with proteins/antibodies/metals, and/or engineering of the nanoparticles able to use the transporters highly expressed on the BBI and neuronal cell surface, used as a Trojan horse [155,157,158], are under evaluation. In neurodegenerative diseases (NDs), one of the additional vital points is the need to counteract redox stress, a primary pathological mechanism in NDs [161]. Interestingly, a pro-oxidant milieu may be an advantage, by “opening” the cargo and allowing the release of the principle on the site of the lesion [162].

In the context of PD studies (animal models), nanoparticle delivery of dopamine and levodopa, ropinirole and apomorphine (dopaminergic agonists), and growth factors (NGF, GDNF) have been tested, reporting positive results in terms of reaching the target, release of the content, good efficacy, and tolerance [156]. Hence, the basis for developing UCB-coated nanoparticles possibly loaded with additional therapeutic factors seems to be a consistent way to explore.

## 5. Conclusions

In the search for a novel therapy for PD, owing to the plethora of the multiple mechanisms involved in the disease, bilirubin is a promising single-therapy candidate accompanied by a sufficient delivery system. Further study regarding bilirubin neuroprotection in PD’s experimental models, which can reproduce clinical PD in humans, is needed.

## Figures and Tables

**Table 1:** Current treatments targeting motor symptoms in Parkinson’s disease.

Treatment	Targets	Clinical Effect	Side Effects/Limitations	References
Pharmacological Treatment				
L-DOPA	Dopamine precursor	Improving motor symptoms	Dyskinesia, nausea, hypotension, muscular rigidity, wearing off effects	[17,18]
Decarboxylase inhibitor (carbidopa, benserazide)	Paired with levodopa to inhibit its peripheral conversion to dopamine	Reducing peripheral L-DOPA side effects: vomiting, nausea, arrhythmia, and postural hypertension		[12]
Dopamine agonists Ergoline-derived agonist (bromocriptine, cabergoline, pergolide, lisuride) Non-ergoline-derived agonist (pramipexole, ropinirole, rotigotine, apomorphine)	Mimicking the endogenous dopamine and stimulating dopamine receptors Binding to dopamine receptor (D1, D2), 5-HT, and adrenergic receptor Specifically binding to dopamine receptor (mainly D2, D3)	Ameliorating motor fluctuations and delaying levodopa administration	Spesific risks of peritoneal, pulmonary, and cardiac/valvular fibrosis Hypotension, impulse control disorder, psychosis, hallucination	[19,20]
Catechol-O-methyl transferase inhibitors (tolcapone, entacapone)	Inhibiting catechol-O-methyltransferase to prevent dopamine degradation	Reducing wearing-off-type motor fluctuations	Nausea, diarrhea, orthostatic hypotension, dyskinesia, risk for hepatotoxicity	[21]
Monoamine oxidase type B (MAO-B) inhibitors (rasagiline, selegiline)	Inhibiting MAO-B to prevent dopamine metabolism	Improving mild symptoms and “off” period	Sleep disturbances, anxiety, nausea, stomatitis, orthostatic hypotension, hallucinations	[20,22]
Anticholinergics (trihexyphenidyl, benztropine)	Antagonism of muscarinic acetylcholine receptor helps to maintain the balance of dopamine and acetylcholine	Mitigating the mild symptoms of tremor and rigidity	Immobilization, urinary dysfunction, gastroduodenal ulcer, depression, epilepsy	[23,24,25]
N-Methyl-D- Aspartate glutamate receptor antagonist (amantadine, memantine)	Enhancing dopamine release and blocks dopamine reuptake	Useful in the control of dyskinesia	Livedo reticularis, ankle edema, confusion, nightmares, withdrawal encephalopathy, and mild peripheral antimuscarinic effects	[12,26]
Non-pharmacological treatment				
Deep brain stimulation	Stereotactic surgery ablations of either the globus pallidus internus or subthalamic nucleus	Improving appendicular motor symptoms (brady/akinesia, rigidity, and tremor), lowering the L-DOPA dose needed, alleviating hyperdopaminergic behaviors, neuropsychiatric fluctuations	Aggravate visuomotor, depressive symptoms, dementia, and surgical complications (intracranial hemorrhage, infections, microlesion)	[27,28]
Cell replacement therapy	Transplantation of hESCs or iPSCs to replace the dopaminergic neuron loss	Under monitoring (ongoing clinical trial phase)	Poor survival of DA neurons, risk of neural tissue overgrowth and neuroepithelial tumors, and could carry mutations	[29,30,31]
